# Supplementary material for: Subjective well-being during the 2020–21 global coronavirus pandemic: Evidence from high frequency time series data
Source: PLoS One. 2022 Feb 16;17(2):e0263570. doi: 10.1371/journal.pone.0263570 (PMC8849501; doi:10.1371/journal.pone.0263570)
Supplement: S1 Appendix — (DOCX) [file pone.0263570.s001.docx]

**Appendix**

**Fig A.1. Validation of the affective life satisfaction measure (ALS) and Cantril scale life satisfaction survey responses.**


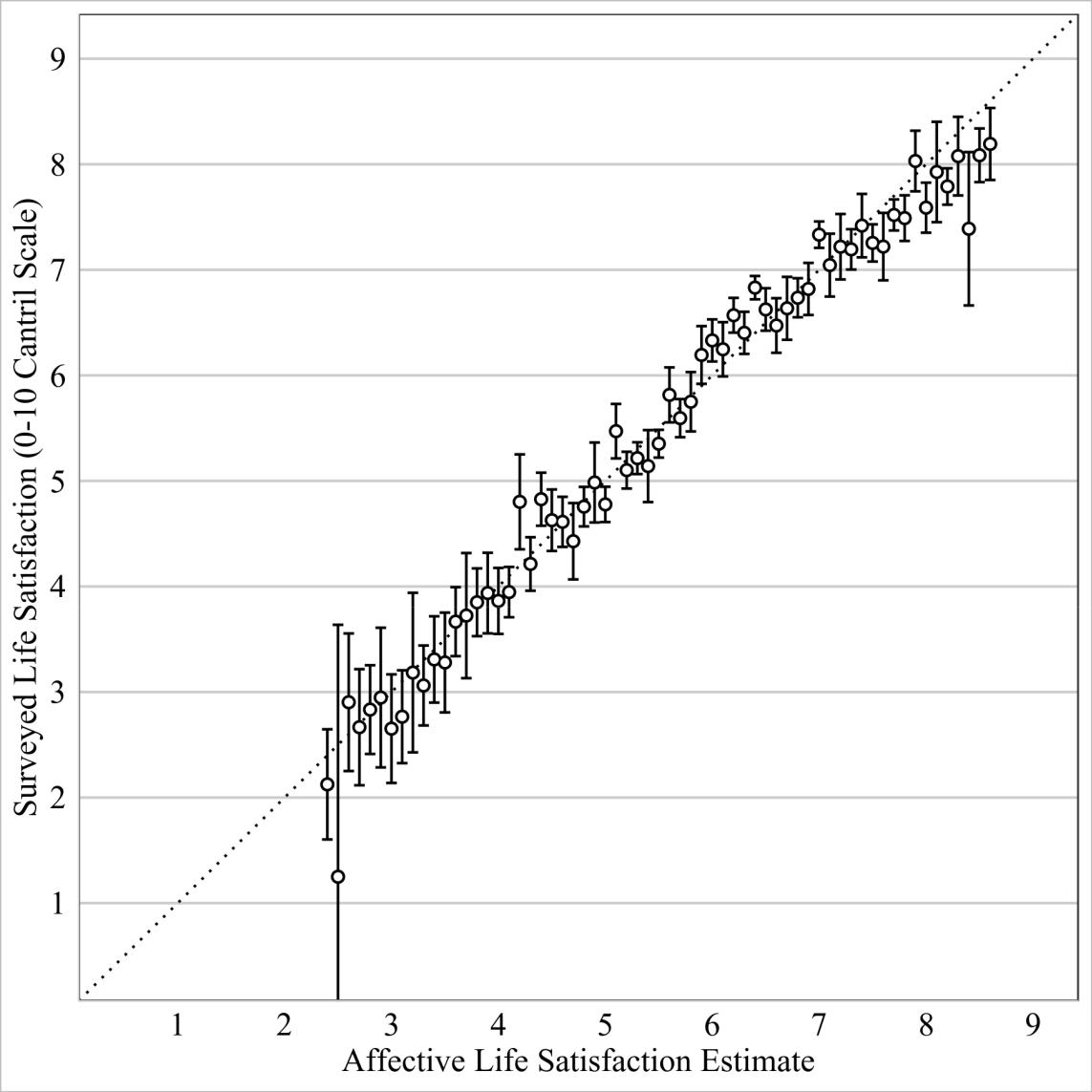


Notes: 45-degree line on the chart indicates perfect equivalence. Mean scores shown by points, and the 95% confidence intervals for clustered observations on the Life Satisfaction Index measure.

**Fig A.2. Validation of the affective life satisfaction (ALS) measure and Cantril scale life satisfaction survey responses, using sociodemographic clusters.**


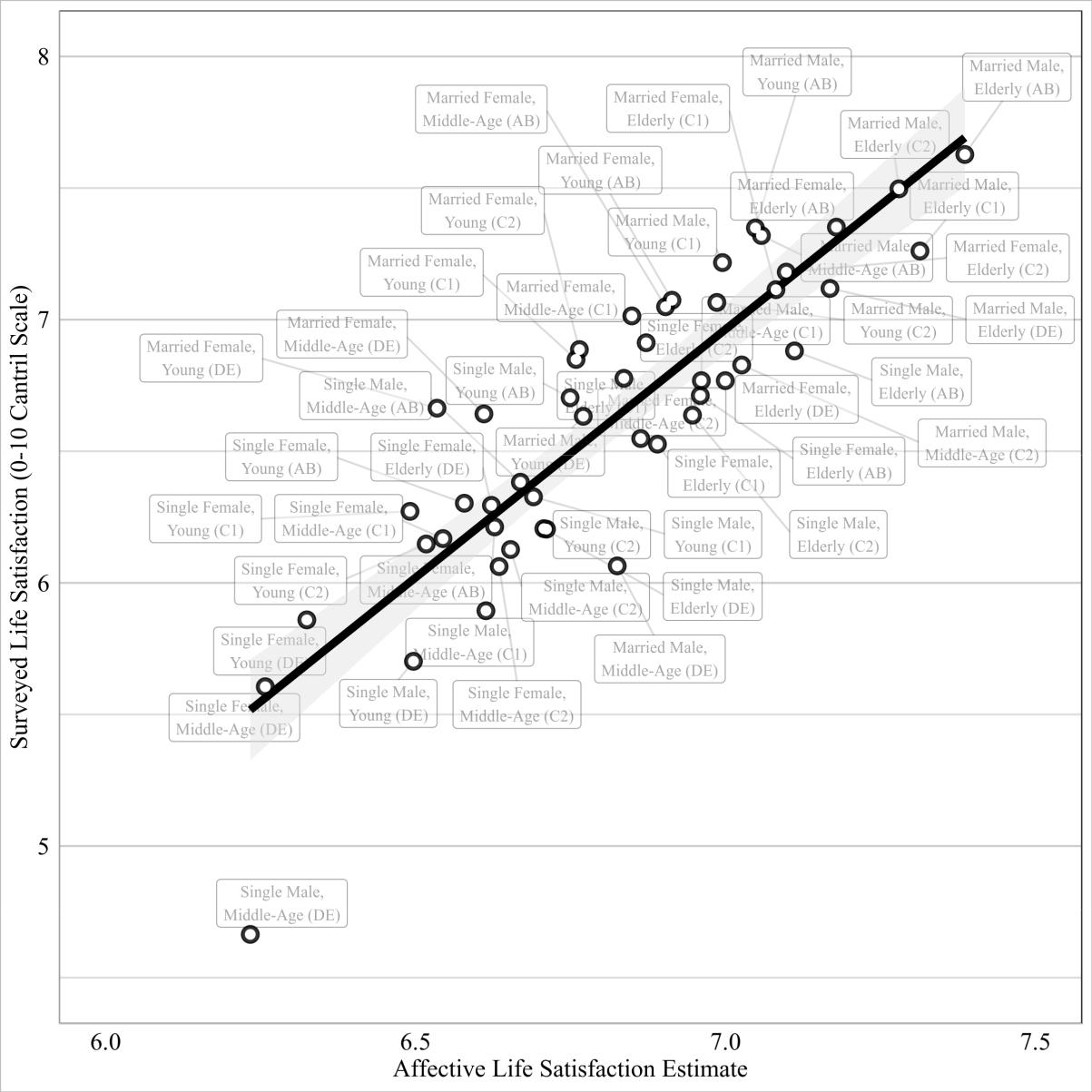


Notes: Mean scores shown by points, with linear OLS line of fit and 95% confidence interval. R = 0.88. Clusters are defined based on aggregates for age, gender, socioeconomic status (social grades A-E), and relationship status (marriage or equivalent relationship vs. single).

**Table A.1. Imputation of the affective component of life satisfaction**

*Dependent variable.*

Life Satisfaction. Cantril Ladder (0-10)

*Positive Affect States*

| Happy | 0.637*** |
| --- | --- |
|  | (0.018) |
| Content | 0.305*** |
|  | (0.017) |
| Optimistic | 0.207*** |
|  | (0.018) |
| Energetic | 0.095*** |
|  | (0.018) |
| Inspired | 0.042** |
|  | (0.018) |
| *Negative Affect States* |  |
|  |  |
| Lonely | -0.347*** |
|  | (0.017) |
| Sad | -0.336*** |
|  | (0.019) |
| Stressed | -0.240*** |
|  | (0.019) |
| Apathetic | -0.175*** |
|  | (0.015) |
| Scared | -0.105*** |
|  | (0.017) |
| Frustrated | -0.096*** |
|  | (0.017) |
| Bored | -0.091*** |
|  | (0.015) |
|  |  |
| Constant | 6.878*** |
|  | (0.017) |
|  |  |
|  |  |
| Observations | 13,954 |
| R^2^ | 0.320 |
|  |  |

Notes: Standardised coefficients. p<0.05; p<0.01; p<0.001.

**Table A.2. Descriptive statistics for multilevel model variables.**

|  |  |  |
| --- | --- | --- |
|  | Sample | Percent |
| Variable | Percent | Missing |
|  |  |  |
| Included as Both Fixed and Random Effect: |  |  |
| Young (18-24) | 8.9% | - |
| Elderly (65+) | 25.8% | - |
| Voted Conservative in previous election | 33.0% | 2.3% |
| Voted Labour in previous election | 29.2% | 2.3% |
| Female | 53.4% | - |
| Social grade - professional/skilled (ABC1) | 59.5% | - |
| Included as Fixed Effect Only: |  |  |
| London | 12.1% | - |
| Southern England (Ex-London) | 33.1% | - |
| Midlands | 16.7% | - |
| Northern England | 24.3% | - |
| Scotland | 8.8% | - |
| Wales | 5.0% | - |
|  |  |  |

Notes: YouGov Weekly Mood Tracker Survey, July 2019 – December 2020. There are no missing observations for age, gender, region or socioeconomic status, as these are already included by virtue of YouGov’s panel sampling methodology.

**Table A.3. Multilevel Models: Fixed Effects**

|  | *Dependent variable:* | | | | |  |
| --- | --- | --- | --- | --- | --- | --- |
|  | Affective Life  Satisfaction | Positive  Affect | | Negative  Affect | | |
| Region: Southern England | 0.019*** | -0.0002 | | 0.003 | | |
|  | (0.005) | (0.011) | | (0.002) | | |
| Region: Midlands | 0.018*** | -0.036*** | | 0.010*** | | |
|  | (0.006) | (0.012) | | (0.002) | | |
| Region: Northern England | 0.006 | -0.054*** | | 0.006*** | | |
|  | (0.005) | (0.011) | | (0.002) | | |
| Region: Scotland | 0.019*** | 0.015 | | -0.002 | | |
|  | (0.007) | (0.014) | | (0.003) | | |
| Region: Wales | 0.011 | -0.050*** | | 0.010*** | | |
|  | (0.008) | (0.017) | | (0.003) | | |
| Female (=1) | -0.072*** | | -0.019** | | -0.045*** |  |
|  | (0.004) | | (0.008) | | (0.002) |  |
| Young (age 18-24) (=1) | -0.014** | 0.279*** | | -0.064*** | | |
|  | (0.005) | (0.015) | | (0.003) | | |
| Elderly (age 65+) (=1) | 0.130*** | 0.050*** | | 0.078*** | | |
|  | (0.005) | (0.009) | | (0.002) | | |
| Voted Conservative in prior election (=1) | 0.097*** | 0.088*** | | 0.037*** | | |
|  | (0.005) | (0.012) | | (0.002) | | |
| Voted Labour in prior election (=1) | -0.044*** | | 0.017** | | -0.038*** |  |
|  | (0.004) | | (0.009) | | (0.002) |  |
| Professional (social grades ABC1) | 0.074*** | 0.187*** | | 0.004*** | | |
|  | (0.004) | (0.008) | | (0.001) | | |
|  |  |  | |  | | |
| Constant | 6.890*** | 1.013*** | | 1.749*** | | |
|  | (0.008) | (0.016) | | (0.003) | | |
|  |  |  | |  | | |
| Observations | 152,041 | 152,041 | | 152,041 | | |
| R^2^ | 0.09 | 0.02 | | 0.07 | | |

Notes: Random effects by period are shown separately. ^†^p<0.1; *p<0.05; **p<0.01; ***p<0.001.
