# Supplementary material for: Subjective well-being during the 2020–21 global coronavirus pandemic: Evidence from high frequency time series data
Source: PLoS One. 2022 Feb 16;17(2):e0263570. doi: 10.1371/journal.pone.0263570 (PMC8849501; doi:10.1371/journal.pone.0263570)
Supplement: Attachment — Submitted filename: Response to reviewers - PLoS One.docx [file pone.0263570.s002.docx]

**Specific Responses to Reviewer Comments**

We would like to thank the reviewers for their time and thoughtful comments as well as the editorial team at PLOSONE for their patience while we have rewritten this manuscript. The original paper was written in the early months of the pandemic, based on the data available at that time, and in response to what was then an emerging research question – whether the recently-implemented lockdown measures were directly negative for mental health and subjective wellbeing. Given the large amount of additional data now available to revisit our earlier hypotheses, we have decided it best to rewrite the article, and the statistical analysis, using the following additional sources:

1. For the Google search data, we now use daily (rather than weekly) data for the entire period from the year preceding the pandemic up until July 2021 for Australia, New Zealand, the United States, Canada, the UK, and Ireland. This comprises a significantly larger number of lockdown episodes, and coronavirus outbreak waves, upon which to test our original hypotheses.

2. For the aggregate-level YouGov Great Britain weekly mood tracker survey, we cover not only the year before the pandemic, but the entire first 18 months following the initial detection of COVID-19 cases in the United Kingdom up until July 2021, which allows us to examine how well successive lockdowns (in November 2020 and the first quarter of 2021) mirrored the pattern of the first such policy response in March 2020.

3. For the individual-level analysis (multilevel models) using the YouGov data we have obtained the source data from YouGov up until December 2020, which now allows us to examine specific group-effect not only during the initial outbreak, but also during the outbreak of the alpha variant in October to December of 2020.

This has been a valuable opportunity to improve the conceptual framing of the paper and its situation within the existing literature – and a full explanation of how we have incorporated reviewer suggestions and addressed reviewer concerns is detailed below.

Inevitably not all the questions raised in this research can be addressed definitively, even with the benefit of additional data and analysis, and we would be glad to revisit these questions in future articles after seeing this work through to publication.

Specific issues addressed in the revised manuscript as listed in the following section.

1. Updating the UK Mood Survey Results to July 2021

In our original article, we had only been able to examine changes in surveyed affect in the United Kingdom in relation to the initial months of the pandemic and the first national lockdown, which occurred in April to June of that year.

With the benefit of an additional year of mood tracker data, we are now able to update this analysis to cover several additional COVID-19 waves: specifically, the increase driven by the spread of the alpha variant in autumn of 2020, the “winter wave” of January-February 2021, and the spread of the delta variant in the summer of 2021. This period also includes one additional major lockdown (from January to April 2021), as well as one “mini” lockdown (in November of 2020).

**Affective Life Satisfaction Index (Top) and Daily COVID-19 Fatalities in the United Kingdom**

The pattern of the initial coronavirus outbreak in the United Kingdom – of an initial drop in wellbeing as the virus spread, reaching a peak at the moment that a national lockdown was announced – occurred both with the spread of the alpha variant in September-October 2020, and the “winter wave” of December 2020 to January 2021. Notably, wellbeing began its decline during the spread of the alpha variant in September and October, long before lockdown measures were eventually announced. A slight dip in wellbeing is also evident at the end of the series with the initial spread of the delta variant – at a time when lockdown restrictions were nonetheless being further eased, rather than increased.

2. Extending the Negative Affect Search Index to 2020-21

Just as we have been able to extend the weekly Great Britain mood tracker survey results until July 2021, we have also similarly extended the negative affect search indexes (based on Google data) to cover the same period for the United States, Canada, Australia, Ireland and New Zealand.

The updated figures are included as below.

| United States   | Canada   |
| --- | --- |
| United Kingdom   | Ireland   |
| Australia   | New Zealand   |

3. Critical Cases: Lockdowns Without Eipdemics, Epidemics Without Lockdowns

A further benefit of the extended observation period is that we now have a much larger universe of observations as regards both national coronavirus waves, and lockdown restrictions imposed in response to the threat of epidemic outbreaks. Critically, whereas the initial months of the pandemic reflect a combination of the first COVID-19 wave with the first effort at mobility restrictions to curtail the spread of the virus, in the following year these two variables have diverged significantly, giving us a much better dataset from which to observe the associations between pandemic severity and related lockdown restrictions. These include both cases in which lockdown measures were imposed early enough to prevent local clusters from developing into national epidemics, and cases in which viral waves were met with either limited policy responses or even a continuation of the easing of prior restrictions.

*a) Lockdowns Without Epidemics*

The key examples of lockdown measures that prevented large-scale viral waves from occurring were in Australia and New Zealand in 2020 and the first half of 2021, where these societies did not experience major national COVID-19 epidemics affecting a significant proportion of their population. The case of New Zealand is especially illustrative, as the country implemented both relatively severe lockdown restrictions on several occasions, and yet, saw exceptionally few cases, as local clusters were resolved before breaking out to the general population.

A comparison of New Zealand and the United Kingdom is shown below. Both countries experienced greater negative affect during lockdown periods relative to the period prior to the onset of the pandemic. Yet in Great Britain, where a nationwide epidemic led to a significant number of COVID-19 fatalities, negative affect spiked to a weekly peak of 4.3 standard deviations above the pre-pandemic mean – an extreme outlier event, which in a normally distributed series, would be situated in the top 0.002% of observations - whereas in New Zealand, the index never rose more than 1.9 standard deviations above the mean.

**Negative Affect Index, COVID-19 Fatalities, and Lockdown Periods: 2020-2021**

| New Zealand   | United Kingdom   |
| --- | --- |

Negative affect in New Zealand and the United Kingdom (top), 2019-2021, plotted by pandemic severity (bottom) and lockdown periods (shaded gray).

*b) Epidemics without Lockdowns*

The alternative example we now have is from the United States, where further coronavirus waves in late 2020 and 2021 were not met with the same degree of economic and social restrictions that were implemented during the first wave in March to May of 2020. During the initial coronavirus wave, a majority of the U.S. population lived in states with declared states of emergency and mandates restricting movement, work and recreational activity. After these orders expired, however, they were not renewed, except in a small number of states, such as California and New York. In contrast to other countries where national and subnational orders were reintroduced, the United States as a whole did not experience any further national lockdown.

During the second “summer wave” of 2020, which was concentrated in southern and western states that had limited policy responses to the pandemic and continued with policy easing throughout this time, negative affect in the United States nonetheless spiked considerably, reaching 2.3 standard deviations above the pre-pandemic mean during this period (in the top 1.1% of the distribution).

4. Lockdowns and Wellbeing - Updating the Findings

One of the benefits of empirical research is that it gives us the opportunity to revisit our hypotheses with the benefit of new data, and revise our views accordingly. As the reviewers note, it is difficult to distinguish the effect of lockdowns and the effect of the pandemic, especially using only data from the pandemic’s initial months, due to endogeneity and high collinearity. With the benefit of an additional year of data, and many “off-axis” cases (lockdowns in response to minor outbreaks, and COVID-19 waves that were met by more limited policy responses), we can have more confidence in our predictions.

As a result of our new data, our key finding regarding lockdowns has altered: we do now find a temporary negative effect on wellbeing associated with the start of a lockdown, *relative to a pre-pandemic baseline*. However, we also find that reversion to baseline is relatively fast (2-3 weeks) and that negative affect falls below the baseline after this point. Examination of the Great Britain mood tracker data also gives a reasonable explanation for why this is the case, as during both extended lockdown periods in the UK (April to June 2020 and January to April 2021) initial increases in sadness, boredom, and loneliness declined after several weeks, while decreases in stress, sadness and fear appear to have been sustained and in the latter two cases, even fell below the pre-pandemic baseline. Meanwhile, by summer 2021 the UK had completely recovered to pre-pandemic levels of overall affect.

**Model Estimated Effects of Lockdown Initiation and Duration**

**Updated Mood Tracker Results, 2020-21**

5. Demographic Effects and COVID-19 Vulnerability

If lockdowns are the key driver of negative affect, then we would expect to find the largest decline in wellbeing among those individuals with the most active social lives, notably in younger age demographics where young people have suffered from reduced contact with friends, social isolation during university studies, and the stress of having to undertake remote learning. By contrast, if it is the pandemic itself which has depressed subjective wellbeing, then we would expect to find this effect most pronounced among clinically vulnerable individuals with a significant risk of mortality from contracting COVID-19. Such fears, moreover, are well-founded: at the time of writing, around 0.2% of the entire population of both the United States and the United Kingdom has died while infected with COVID-19. In both cases, the large majority of fatalities concentrated among the elderly, for whom the population fatality rate is therefore considerably higher.

Multilevel results for the remainder of 2020 show findings consistent with the hypothesis that concerns regarding the risk posed by COVID-19, and not lifestyle changes imposed by lockdown, explain the shifts in affective wellbeing. The significantly negative coefficients for weekly period effects among the elderly indicate that, over and above the general effects detected in the population as a whole, wellbeing was specifically depressed among the elderly by around 50% greater than in the population as a whole; meanwhile, no significant deviation from the national average is found in the younger (18-24) population. It is also notable that in late 2020, when coronavirus cases were rising long before the re-implementation of lockdown measures in November, the mood of elderly respondents was again depressed below the baseline.

This decline in affect among the elderly population relative to the general population is also visible from descriptive trendlines drawn from the dataset, as shown in the figure below which plots the lead of elderly wellbeing over that of the non-elderly population. Prior to the onset of the pandemic, affective life satisfaction among the elderly was +0.2 higher than the general population. With the spread of COVID-19 in February and March of 2020, this fell to +0.1, before recovering back to its initial lead by the end of the first lockdown. A further small dip then occurred later in the year, concurrent with the spread of the alpha variant throughout the UK.

**Lead in Affective Life Satisfaction of the Elderly (65+) Relative to General Population**

6. Testing the Reliability of the Google Negative Affect Search Index

One concern with our earlier work related to whether the mapping of surveyed mood states (from weekly nationally representative YouGov surveys) to affective state term searches on Google was overfitted to the data, and hence would not correlate to the same degree in out-of-sample tests (e.g. future data). This was also a concern that we felt at the time, given the particular nature of the period under consideration, and the possibility that the relationship between these variables for the July 2019 to July 2020 period might not have endured beyond this point.

With the benefit of an additional year of data, we are now able to present results showing how the original methodology has fared over time.

The original chart showing their relationship is given by the figure below:

**Original Test (July 2019 to June 2020 data, smoothed):**

Which we can now update to show the trendline in the two variables, using the same methodology, for the out of sample period in the year following the development of the negative affect index (from the YouGov tracking survey) and the negative affect search index (from Google search data):

**Updated in-sample and out-of-sample comparison (no smoothing):**

While there has been a very slight decline in tracking accuracy between the two measures, it is small enough to reassure us that the methodology of inferring country-level negative affect for a wider sample of countries based on search index data remains robust.

**Editorial Requests**

**Comment 1. The main result cannot be interpreted as it is now, namely as the causal effect of lockdown on wellbeing, as a separated one from the general effect of the pandemic. Furthermore, they both strongly recommend to tone-down the claim of causality. In the light of this the authors might also want to reconsider the title to some extent.**

Response to comment 1. We have given the piece a new title to reflect what is now its primary contribution, namely, a) to provide detailed descriptive information, via high frequency survey and online search data, of mood state trends during the first two years of the pandemic, and b) to allow for predictions regarding their fluctuation in line with pandemic waves and lockdown measures. We are of course open to alternative title suggestions.

**Comment 3. Please ensure that your manuscript meets PLOS ONE's style requirements, including those for file naming. The PLOS ONE style templates can be found at https://journals.plos.org/plosone/s/file?id=wjVg/PLOSOne_formatting_sample_main_body.pdf and https://journals.plos.org/plosone/s/file?id=ba62/PLOSOne_formatting_sample_title_authors_affiliations.pdf**

Response to comment 3. We have done our best to conform to the style guidelines. Thank you for the links.

**Comment 4. We note that you have indicated that data from this study are available upon request. PLOS only allows data to be available upon request if there are legal or ethical restrictions on sharing data publicly. For more information on unacceptable data access restrictions, please see http://journals.plos.org/plosone/s/data-availability#loc-unacceptable-data-access-restrictions. In your revised cover letter, please address the following prompts: a) If there are ethical or legal restrictions on sharing a de-identified data set, please explain them in detail (e.g., data contain potentially sensitive information, data are owned by a third-party organization, etc.) and who has imposed them (e.g., an ethics committee). Please also provide contact information for a data access committee, ethics committee, or other institutional body to which data requests may be sent. b) If there are no restrictions, please upload the minimal anonymized data set necessary to replicate your study findings as either Supporting Information files or to a stable, public repository and provide us with the relevant URLs, DOIs, or accession numbers. For a list of acceptable repositories, please see http://journals.plos.org/plosone/s/data-availability#loc-recommended-repositories. We will update your Data Availability statement on your behalf to reflect the information you provide.**

Response 4. A replication dataset containing the daily negative affect search index scores for six countries from 2019-2021, together with independent variables used in the analysis, will be uploaded with the final submission, in addition to the YouGov weekly mood tracker data.

**Comment 5. Please ensure that you refer to Figure 2 in your text as, if accepted, production will need this reference to link the reader to the figure.**

Response 5. Done.

**Comment 6. We note a previous version of your study was published by Bennet Institute for for Public Policy:**

[**https://www.bennettinstitute.cam.ac.uk/media/uploads/files/Happiness_under_Lockdown.pdf**](https://www.bennettinstitute.cam.ac.uk/media/uploads/files/Happiness_under_Lockdown.pdf)**.**

**Please kindly clarify the following points: a. Please clarify if the Bennett Institute for for Public Policy article was peer reviewed. b. Please also clarify if the previously published article has been copyrighted. For your reference, PLOS ONE publishes all content under a CC BY 4.0 license (https://creativecommons.org/licenses/by/4.0/) which means that all material on our website is freely available online, and any third party is permitted to access, download, copy, distribute, and use these materials in any way, even commercially, with proper attribution. If the figures or text have already been published and copyrighted, authors must provide proper attribution, referencing the source clearly, and obtain permissions if the content is copyrighted.**

Response 6. Having made significant changes since our initial research, we would like this article to be treated as a separate and supplementary contribution to our earlier work. The manuscript submission is not copyrighted, and the figures in the body of this revised version have not previously been published.

**Reviewer 1**

**Comment 1. the authors should reshape their presentation and discussion of findings (the key exhibits should be in the main text and the remaining ones in the appendix)**

We’ve have substantially rewritten the text and tried to be mindful of this comment. If the reviewer thinks there are any exhibits in the new version that should be in appendices, please name them.

**Comment 2. Causality vs. predictability: I have to say that I am totally baffled by statements such as “We are able to separate the effects of the pandemic from those of the lockdowns by utilising weekly data […]”. The lockdown does not occur in a vacuum, but as an endogenous response to an “exogenous shock”, the pandemic. Without clear assumptions stated either graphically (e.g. via DAG) or in equations (e.g. via structural models) is very difficult to assess how such an endeavour is even possible (https://journals.sagepub.com/doi/full/10.1177/2515245917745629). Given the way the authors present their findings, a natural question is: Should we expect a positive effect in the case of a “lockdown” without a pandemic?**

**Identification of causal effects: To claim that one can separately identify the effect of the “lockdown” (D) on subjective wellbeing (Y) from the effect of the “pandemic” (Z) seems a very strong, incredible claim. Any identification of causal effects must rely on assumptions.**

**One can identify the effect of the “pandemic” (Z) using a before-after strategy so long as seasonal effects accounted for (e.g. comparing Y before March 2020 vs. post March 2020 against before March 2019 vs. post March 2019); this is a “difference-in-differences” (DID) strategy with two time dimensions (year and month). The key assumption is that there are no other confounding factors changing in between periods (e.g., “parallel trends assumption”): in other words, the average change in Y would have been the same between pre-March 2019 and post-March 2019 that the one between pre-March 2020 and post-March 2020 in the absence of the pandemic. Unfortunately this paper does not have YouGov data before June 2019 (this is an important limitation of this paper).**

**But things are more cumbersome if one wants to identify the effect of the lockdown (D). For one thing, D is clearly a response to Z, so I wonder how one can identify the effect of D separately from the effect of Z. While one can think of Z as an exogenous shock, and try to get rid of seasonal effects via DID, D is an endogenous response to the pandemic. In this regard, it seems to me that the best one can hope for in this setting is to use Z as an instrumental variable for D and assume that the exclusion restriction is satisfied, i.e., that Z affects Y only via D. Effectively, then, one can at most think of identifying the effect of D thanks to Z. In practice, however, one will need to relax the exclusion restriction (e.g., https://www.jstor.org/stable/41349174)**

**In any case, if one is interested in causal effects, and disentangling causal effects, assumptions must be made, carefully stated, and discussed. This manuscript does not provide such causal inference approach, and it belongs to the realm of prediction studies.**

**Predictability (correlational) exercise: Do lockdowns predict subjective-wellbeing? One needs to be crystal clear about the fact that predictability is a complete different thing than causal inference (e.g. umbrellas don’t cause rain). I think the authors of this manuscript should be very careful in the way they present their findings and their interpretation of the empirical analysis in light of existing work. In general, the fact that a correlational analysis shows a strong positive relationship does not tell much, if anything at all, about the sign of the causal relationship between two variables. Moreover, things are quite complicated in the present setting, where there are important dynamics and different groups which generate a host of heterogeneities: the severity of the pandemic varies over time; its impact varies by time and (demographic) group; and the impact of the lockdown varies by time (or is a function of time) and (demographic) group, too.**

**The sentence “Alas, most empirical studies … of the pandemic” is confusing, since it seems the study will focus on causal effects, while indeed it will focus on correlations: “Were lockdowns associated with an improvement or worsening in subjective wellbeing?” Whether this is “essential input into contemporary and future policy debates” is debatable.**

**While the paper is in generally well written, there are a few paragraphs that generate a bit of confusion. On page 4 the authors write: “A shortcoming of these studies is an inability to distinguish empirically the effects of the pandemic from the effects of lockdown policies […] this confounds the effects of the two events. It is important that we identify the effects of lockdowns independently”. However, on page 2 the authors stress that “our results are primarily descriptive rather than causal”, so both the previous critique to existing work and arguing that “it is important that we identify the effects of lockdowns independently” are frankly problematic.**

**Many of the existing papers on the pandemic and mental wellbeing are well suited to identify the causal effect of the pandemic (under more or less explicit assumptions). However, the current paper claims to be descriptive and at the same time be concerned about papers which are able to identify well-defined causal effects (pandemic – including lockdown – effects). This requires a diligent revision and reformulation.**

We take the reviewer’s point regarding causal vs *predictive* language, and have tried to rephrase the paper accordingly. We thank the reviewer for clarifying exactly what is at issue. Our intention is to say that we would predict subjective well-being to improve following a lockdown *in response to pandemic outbreak*. We have outlined these issues in a few places in the text, including the challenge posed by the endogeneity of lockdowns to pandemic outbreaks.

**Comment 3. Page 3 does not seem to provide a thorough review of existing work, omitting relevant references, of published (and unpublished) studies trying to understand the effects of the COVID-19 pandemic and the lockdown on mental wellbeing. While some papers focus explicitly on lockdowns, many others acknowledge that they cannot disentangle between the effects of the pandemic and the lockdown. The following studies seem relevant to the present paper:**

**Pandemic and mental health:**

**• Daly M, Sutin AR, Robinson E. Longitudinal changes in mental health and the COVID-19 pandemic: Evidence from the UK Household Longitudinal Study. Psychological Medicine.**

**• Daly M, Robinson E. Psychological distress and adaptation to the COVID-19 crisis in the United States. Journal of Psychiatric Research.**

**• Davillas A, Jones AM. The COVID-19 pandemic and its impact on inequality of opportunity in psychological distress in the UK. ISER Working Paper Series 2020-07.**

**• Ettman CK, Abdalla SM, Cohen GH, Sampson L, Vivier PM, Galea S. Prevalence of Depression Symptoms in US Adults Before and During the COVID-19 Pandemic. JAMA Netw Open.**

**Pandemic and mental health by ethnicity:**

**• Proto E, Quintana-Domeque C. COVID-19 and mental health deterioration by ethnicity and gender in the UK. PLoS ONE.**

**Pandemic and mental health by gender:**

**• Etheridge B, Spantig L. The gender gap in mental well-being during the Covid-19 outbreak: Evidence from the UK. Covid Economics 33.**

**• Oreffice S, Quintana-Domeque, C. Gender inequality in COVID-19 times: Evidence from Prolific participants in the UK. Journal of Demographic Economics.**

**Lockdowns and mental health:**

**• Adams-Prassl A, Boneva T, Golin M, Rauh, C. The Impact of the Coronavirus Lockdown on Mental Health: Evidence from the US. Human Capital and Economic Opportunity Working Group.**

**• Banks J, Xu X. The mental health effects of the first two months of lockdown and social distancing during the Covid-19 pandemic in the UK. Covid Economics 28.**

**• Niedzwiedz CL, Green MJ, Benzeval M, et al. Mental health and health behaviours before and during the initial phase of the COVID-19 lockdown: longitudinal analyses of the UK Household Longitudinal Study. Journal of Epidemiology & Community Health. 2020.**

Response 3. Thank you for these helpful suggestions. We have incorporated several of them into the article. Many used the same data set – the household longitudinal study (HLS) from the UK. We had previously included a paper by Banks and Xu in our literature review using this study, but that was an IFS working paper. To avoid duplication, we’ve focused on citing the published, peer-reviewed papers that use the HLS.

**Comment 4. There are two key issues which are important in interpreting the results and which require additional work: compositional effects and anticipation (overshooting) effects.**

**The first relates to compositional effects. The data the authors use is cross-sectional, not longitudinal. This opens the possibility that the lockdown has a mechanical effect on the type of respondents. With the YouGov data it is possible to look at a bunch of demographic characteristics. The authors should plot the mean of different demographic characteristics of the respondents over time and look for patterns.**

**The second is about anticipation (or overshooting) effects. To what extent the correlation captured is not just reflecting an anticipation effect? In other words, people anticipate that a lockdown is coming, and the closer to the lockdown date the lower the reported mood (the higher Negative Affect) is. Indeed, very tough lockdowns were implemented in Italy (9 March 2020) and in Spain (13 March 2020). The earliest lockdown analysed by the authors is the one in the US (21 March 2020). Perhaps once people understand that the lockdown is not as tough as the ones in Spain and Italy, then mood improves. This is a different explanation for the findings documented in this paper, and requires further clarification and discussion.**

Response 4. The respondents in each week of the YouGov tracker are drawn from a nationally representative sample by age, gender, social grade, and region, hence the percentages do not vary from one week to the next. Moreover selection in to the survey is not dependent on the respondents, but on YouGov, who select participants in their total UK panel (of more than 1m individuals) to be part of the circa 2000 that are given the Mood tracker survey (rather than a different survey) to complete each week. In this sense, we do not anticipate sample bias being an issue from one week to the next.

Regarding anticipation effects, it is a reasonable hypothesis that respondents in February or March of 2020 might have anticipated the coming pandemic, and this would explain why wellbeing began to dip among e.g. vulnerable groups at risk of mortality (such as the elderly) before the actual fatality rate rose. With the benefit of a full time series from 2020 to late 2021, we now also see a similar pattern with future waves, and some signs of attenuation this year after successful vaccine rollouts have reduced the fatality risk. In September-October 2020 we can see this effect as the alpha variant began to spread in the UK, for example. It is possible that there was anticipation of lockdown, but having served as a government consultant during this period, even within government there was uncertainty regarding the eventual policy response and whether a further lockdown would be announced, making it less plausible that the general public could have anticipated either the nature or the duration of future mobility and workplace restrictions until very shortly before a decision had been reached.

**Comment 5. The authors should use alternative measures of the severity of the pandemic. Cases depend on whether widely testing was available (which varied across countries, in particular, early in the pandemic). The authors should present their analysis using deaths (instead of cases) too. Of course, there are also measurement issues with deaths, but it is another complementary way of looking at the severity of the pandemic.**

Response 5. We have switched in the current manuscript to using COVID-19 fatalities rather than diagnoses, as differences in testing regimes over time render the diagnoses data unusable when making comparisons over the course of the full 2020-2021 period. While the COVID-19 fatalities data also is affected by changes in testing efficacy, this is the case to a significantly lesser extent due to the practice established early in the pandemic of conducting post-mortem COVID-19 testing, which ensures that only a low rate of fatalities caused by coronavirus manage to escape official reports, at least in the countries included as part of the analysis (the US, the UK, Ireland, Australia, New Zealand and Canada).

**Comment 6. Lack of data**

**It is unfortunate that the authors cannot replicate Figures 1 and 2 with data for January-June 2019 to account for seasonality effects (since the YouGov data are only available from June 2019). Nevertheless, and for completeness, it would be interesting to plot the data from June 2019 to June 2020.**

Response 6. With the benefit of the extended series (to July 2021) it is now possible to estimate seasonal effects based upon two years of data (2019-2021), both for the UK and on average across the countries in the multi-country sample. Monthly dummy variables were included as controls in the time series models and display a clear seasonal pattern of rising subjective wellbeing from April to August, followed by depressed affect from October to March.

**Comment 7. Regression analysis**

**In addition to adding the analysis using deaths rather than cases, I think it is important to display the following set of results:**

**• Not controlling for lagged NA**

**• Controlling for country FE**

**• Controlling for month FE**

**• Interacting “days in lockdown” * severity of pandemic**

**• Interacting “days since easing of lockdown” * severity of pandemic**

**What is the interpretation of the coefficient on “days since easing the lockdown”? Is easing the lockdown a bad thing?**

Response 7. We have incorporated almost all of these suggestions in to the models used in the revised manuscript:

1) Models are included both with (Models 2-3 and 5-6), and without (Models 1 an 4) the lagged dependent variable.

2) Country fixed effects are now included for all models.

3) Month fixed effects are now included for all models.

4) A variable for days in lockdown times severity of pandemic is now included in Models 3 and 6.

We agree that the interpretation of days under lockdown is unclear, in the absence of a general dummy variable for whether the country is in a lockdown period or not. In the revised models, we now include both this dummy variable (L) and a (logged) days under lockdown variable (LD), which has the following intepretation: when LD is zero (at the start of the lockdown), L tells us the immediate impact on SWB at the outset. We then assume a decay of this effect over time, which is given by the coefficient LD. In the model results, L is positive and LD negative, implying that entering a lockdown results in an initial increase in negative affect, which then declines to zero after a number of days. The use of a log function entails an implicit assumption regarding non-linearity of the decay until a new steady state (of SWB) is reached.

**Comment 8. Other demographic characteristics**

**Do the authors have information on ethnicity and key worker status? I think it would be really interesting to see an analysis along these characteristics in Figure 8.**

Response 8. Regrettably, this information is not available from either YouGov or Google. While ethnicity data was included in the YouGov survey that we obtained in May 2020, it does not form part of the December 2020 file now used in our analysis. In our earlier analysis using the May 2020 data we found no significant effects associated with ethnic minority status, beyond the general sociotropic effect affecting all groups in society.

**Comment 9. Data availability. The authors write: "The raw data cannot be shared publicly as they are the commercial property of YouGov. However, the time series of the data is published weekly here and is sufficient to verify our analysis: https://yougov.co.uk/topics/science/trackers/britains-moodmeasured-weekly. We will make all do files available to any researcher who wishes to replicate our analysis." However, the link "https://yougov.co.uk/topics/science/trackers/britains-moodmeasured-weekly" does not seem to be working.**

Response 9. The correct URL is https://yougov.co.uk/topics/science/trackers/britains-mood-measured-weekly. We thank the reviewer for bringing this to our attention.

**Comment 10. Minor comments**

**• Page 4, paragraph 1: it is not Good Health Questionnaire, but General Health Questionnaire.**

**• Page 4, paragraph 2: heterogeneous effects by demographic CHARACTERISTICS.**

**• Page 10 (OECD 2013). –space needed—All**

**• Figure 7: Does the lockdown starts when the line changes from “black” to “white” or is this indicated by the vertical line?**

**• There are 8 countries and 50 weeks of data: 400 observations. Column 5-6 display 376 observations, so 47 weeks of data are being used?**

**• Clustering standard errors with 8 countries is problematic. Wild Bootstrap should be used to ensure statistical inference with desirable properties: https://journals.sagepub.com/doi/full/10.1177/1536867X19830877**

**• Should the equations contain error terms?**

Response 10. Many thanks for these comments and suggestions, which we have incorporated.

**Reviewer 2**

**Comment 1. It is not possible to accept the main argument that the paper distinguishes lockdown effects from the pandemic. The analyses are very rigorous but they cannot do much about the mere fact that lockdowns were introduced (and will always be introduced) in response to a crisis and therefore, there are no effects of lockdowns without the effects of pandemic. The results, therefore, are better interpreted as showing that the lockdowns attenuate some of the negative changes in mood that occurred during the pandemic, most plausibly by providing a sense of security and safety. This interpretation is supported by this basic intuition, as well as the main results reported in the paper which shows a rise in mood that follows from a reduction in mood. This interpretation is also supported by the corollary finding “that the seeming effectiveness of lockdowns in improving mood is conditional upon pandemic severity.” The authors also acknowledge this idea somewhere in the discussion by stating “ lockdowns improve SWB by ameliorating stress and fear associated with pandemic outbreaks. If there is no serious viral outbreak and thus little stress and fear to ameliorate, then lockdowns won’t improve SWB.” These arguments should be more central to the paper and dictate the title, abstract, and intro.**

Response 1. This comment echoes those of reviewer 1. As we discussed in our responses there, we have endeavored to remove any trace of a causal language from our paper and make only predictive claims. This includes removing language about “distinguishing” the impact of the pandemic from those of lockdown or identifying them independently.

Our analysis is now also stronger and clearer as we have now extended the range of the data by a full year (from July 2020 to July 2021). As a result, we are now able to examine cases in which lockdowns occurred without local clusters becoming national epidemics (New Zealand and Australia in 2020), and cases in which coronavirus waves occurred with either minor policy restrictions or continued policy easing (e.g. the most recent delta wave in the United Kingdom, or the summer second wave across the south and southwest of the United States in 2020).

With the benefit of a much-expanded range of lockdown instances across the countries included in our analysis, we have updated our conclusions in line with our new findings, in that we do now find a small negative affect increase when lockdowns are introduced, but that this declines fairly rapidly before switching to a below-baseline level of negative affect. The same caution regarding effect interpretation here, of course, still applies: for example, it could be that the brief negative affect identified at the start of lockdown is due to a rise in COVID-19 cases that has not yet fed through to a rising fatality rate.

**Comment 2. The authors should consider referring to ‘mood’ as opposed to ‘subjective well-being’ as this more accurately represents their data. Mood is a critical component of SWB and this link can be emphasized by citing literature and correlations with the life satisfaction measure but given that both of their outcomes measure mood (with some links to life satisfaction in one of the data), the paper seems to study mood and this should be reflected in the title, abstract, text etc. Relatedly, the efforts to link mood data to life satisfaction in the Yougov analysis doesn’t seem convincing. Using the lower quality life satisfaction data and so much imputation and complication doesn’t seem to be justified. Why not just use a simple index for positive and negative moods, and a composite mood index? The authors already use a negative affect index from Yougov data in their follow-up analysis and show trends that support their main finding in Figure 4, why not use this index (in addition to positive mood) in the main analysis too?**

We have altered the discussion of our methods and results to refer more explicitly to affect rather than SWB. Our literature review is broader, so we discuss SWB, mental health, and other related items there, but once we turn to our results we stick to affect. We have focused on SWB in the title because our discussion and results should be of interest to SWB scholars broadly, and they are less likely to find the paper if the title only mentions mood.

Respectfully, we do not agree that we should simply use a composite mood index as this would imply attaching equal weights to each mood. This would amount to saying that happiness is on par with boredom, for example, which is not intuitively appealing. Of course, arbitrarily attaching our own weights to different mood states is also not appealing. Using life satisfaction to weight the mood states’ contribution to our affective life satisfaction index is preferable because it allows respondents to assign the weights. We note that some mood states are much more heavily weighted. For example, happiness accounts for 24% of the total variation whereas boredom only accounts for 3%. More broadly, the mood items that improve during lockdown are typically the ones that explain relatively larger portions of the variance in the weighted index, whereas the items that deteriorate explain relatively little of the variance. A uniformly weighted index would thus misrepresent what moods matter most to people. We have inserted this justification into the text of the paper.

**It is very important that the short timeframe for the estimates and the patterns in data that follow after this short-frame (how the data looks after the 1 month) are reminded to the reader in the abstract and throughout the manuscript. These are patterns observed early on during the lockdown and they mostly seem to disappear later on. Can the Figures 3 and 4 show later time periods in the graph to indicate this?**

Response 3. We have now expanded the range of the data to include the entire first 18 months of the pandemic, from February 2020 to July 2021 (as well as the period before the pandemic).

**Comment 4. The negative affect trend in Figure 2 seems to indicate the complete opposite of the main findings throughout the manuscript. There are striking increases in boredom, loneliness, and apathy after the lockdown. This is very important and directly contradicts the main arguments in the paper. On the other hand, the reductions in stress and scare are very relevant and support the main mechanism that the lockdown increases a sense of security. Making these more central could enrich the theoretical contributions of the paper, which is currently not very strong. A follow-up question is: does this discrepancy in mood items emerge in the Google data too (i.e., results are different for stress, scare vs. boredom etc.) ? No matter if it does replicate in Google data or not, these diverging results need to be discussed very explicitly, they are important to acknowledge and they even help explain the results.**

An excellent point. We have adapted the discussion of the raw mood trends and our motivation of the affective life satisfaction index to address this comment. We discuss each mood state individually, noting that the heterogeneous trends suggest that lockdowns ameliorate the negative effects of the pandemic on mood but also have their own pernicious side effects. We also explicitly state that, taking all negative mood states together, negative affect rises with pandemic onset and then continues rising, albeit more slowly, after the implementation of lockdown (in the YouGov data). This is one part of the motivation for the affective life satisfaction index – to get some sense for which mood states matter more to people. .

**Comment 6. The causal language needs to be removed from the manuscript. Although the authors admit they are estimating associations, there is a heavy use of the words such as ‘effects’ or ‘impacts’. These words are better omitted.**

We hope we have addressed this issue to the satisfaction of both reviewers..

**Comment 7. The discussion on Sweden in the intro is better placed in limitations.**

We have simply cut this out and included more general points regarding the difficulty of causal inference in this case. This helps to keep the paper streamlined and straightforward.

**Comment 8. I recommend that the following work on the topic and other recent studies that may have come out during the last months are integrated into the introduction and that the findings are discussed in light of this evidence:**

**Aknin, L., De Neve, J. E., Dunn, E., Fancourt, D., Goldberg, E., Helliwell, J., ... & Amour, Y. B. (2021). A review and response to the early mental health and neurological consequences of the COVID-19 pandemic.**

**Giurge, L. M., Whillans, A. V., & Yemiscigil, A. (2021). A multicountry perspective on gender differences in time use during COVID-19. Proceedings of the National Academy of Sciences, 118(12).**

**VanderWeele, T. J., Fulks, J., Plake, J. F., & Lee, M. T. (2021). National well-being measures before and during the COVID-19 pandemic in online samples. Journal of general internal medicine, 36(1), 248-250.**

Thanks for these citations. We have integrated them into the literature review along with the suggestions of reviewer 1, above.

**Comment 9. "Underemployed men saw a marked increase in their SWB during lockdown" this could also be because of norm effects as more people become unemployed, unemployment could hurt less because there is less stigma, identity effects**

Agreed. However, we have now removed this whole section from the manuscript.

**Comment 10. Please temper the positive picture argument here “While our results paint a positive picture of the impact of lockdowns on SWB, “**

We have deleted this sentence.

**Comment 11. Please provide more justification and explanation about why restricting sample space is necessary and what it entails “rising to -17% in models where the sample space is restricted to the period following lockdown onset (Models 3-4).” It is unclear what the sample in this sentence is.**

As our results have changed with the wider sample in the revised paper, we have deleted the sentence in question. However, we still need to provide a justification for using two sampling windows. As such, we have inserted the following explanation to the section on model specification:

Using two sampling windows in this way illuminates how the association between lockdown and mood differs statistically depending on whether it is assessed relative to a pandemic-free world (models 1-3) or to a world with pandemics but no lockdown (models 4-6).

**Comment 12. The authors can be more accepting of the limitations of the cross-sectional nature of the data and present more information and discussion about the representativeness of both samples and whether and how the sample composition may have changed over time. This doesn't invalidate the importance of the findings but it is important to acknowledge and report in a study who has such strong population-level policy implications.**

Response 12. We have included a discussion of this issue in the limitations section. However, we stress that the sample composition is nationally representative in each survey week, controlling for age, gender, socioeconomic status and region. These percentages are broadly stable for the entire duration of the survey.

**Comment 13. Are there any descriptive statistics that show how prevalent the specific population groups are in the data?**

**How is controlling for lagged values of the outcomes tackling hedonic adaptation? Please explain the mechanics/rationale for this new approach.**

This strategy was recommended to us by Andrew Oswald, who wrote many of the seminal papers on adaptation. We have tried to provide a longer explanation in the revised version of the paper. The modelling strategy relies on a difference equation framework. Suppose:

$Y_{t}={\alpha Y}_{t-1}+w_{t}$

Where Y is subjective wellbeing, w is vector of variables like pandemic severity, lockdowns etc., and t subscripts refer to periods of time. Wellbeing in the current period is then a function of wellbeing in the previous period plus shocks in the current period. If adaptation is rapid and complete then α = 0, which means that wellbeing becomes only a function of shocks in the current period. On the other hand, if α = 1, then there is no adaptation and wellbeing is comprised of its previous value plus new shocks. In our results, we find that α = 0.2 (i.e. the beta coefficient on the lagged term is 0.2). Given that we are using a 2-week lag, this suggests very fast but not complete adaptation as only a fifth of the value from the previous period is carried over into the present period.

**Comment 14. The negative trend in Figure 4 graph doesn’t match the results for Figure 2 ‘negative affect average’. Please acknowledge and/or reconcile this discrepancy.**

Response 14. This arose from the method used to calculate the averages for the mood states in the previous Figure 2 that was submitted, which calculated slopes for each of four periods (pre-pandemic, COVID-19 outbreak, the first month of lockdown, and the subsequent period) and took the last average as the intercept for the new period. With the benefit of substantially extended observational data (from 2019 to 2021), we no longer use these breaks and instead simply show the rolling averages ove r time, making the presentation of Figures 2 and 4 consistent.

**Comment 15. The paper goes back and forth with the data & analysis match, starting with YouGov data, then cross-country data, and then time-series analysis in cross-country data, and then finishing off with subgroup analysis in YouGov data again. It could simplify the paper if the authors start with the cross-country data (it only shows negative mood anyways) and time-series in this data, and then finish up with the Yougov analysis on negative and positive mood+subgroup analysis.**

Respectfully, while we agree that the back and forth with the data sources is not ideal, we think there are compelling reasons for our choice of structure. In particular, we start with the YouGov data because it is our highest quality data. We then validate the cross-country data using the YouGov data. The cross-country analysis is a check to see whether the trends we observe in the high quality data seem to play out more generally. If we led with the cross country analysis we would still need to introduce the YouGov data first for validation purposes, so the back-and-forth structure would remain.
