# Supplementary material for: Subjective well-being during the 2020–21 global coronavirus pandemic: Evidence from high frequency time series data
Source: PLoS One. 2022 Feb 16;17(2):e0263570. doi: 10.1371/journal.pone.0263570 (PMC8849501; doi:10.1371/journal.pone.0263570)
Supplement: Attachment — Submitted filename: Response to Reviewers, 2nd RR.docx [file pone.0263570.s003.docx]

**Response to Reviewers, 2^nd^ R&R, PLOS One**

We thank the reviewers for their thoughtful and manageable comments and suggestions, which we have tried to address to the best of our ability. We are particularly grateful for reviewer 2’s emphasis on further discussion rather than additional analysis. Please see an itemised list of our changes and responses below.

REVIEWER #1

**I would suggest proofreading the article one more time for typos. For instance, on page 32, instead of . you should probably use : after "to control for":**

Thanks, yes there were quite a few typos around the place.

REVIEWER #2

**1 - The initial spike in negative affect at the onset of the lockdown…it would be difficult to ask for more analysis to explain this. At least, the authors can include these in the discussions – how do authors explain this spike?**

This is a good question. We have added new content to the paper on pg. 26 to provide some discussion, specifically:

“It is unclear why negative affect is above zero initially. It may simply be because deaths are spiking around this time. Or it could be an anticipation effect. Respondents might expect more deaths if the pandemic is severe enough to warrant a lockdown. They may also expect the experience of lockdown to be very unpleasant. As the reality of lockdown emerges, both in terms of its effect on death rates and its liveability, negative affect declines.”

**2 - There is a very good discussion of the mechanisms in the literature review, but the authors don’t come back to this to discuss their findings… the changes in death rates and the affect seem to mirror one another in all countries, which suggests that the declines in negative affect may be related to lockdowns stopping deaths. There are probably other papers showing this – that the lockdowns reduced Covid-related deaths?** **May be important to cite in the discussion.**

A good suggestion. Citations 1 & 2 refer to papers in *Nature* showing a decline in excess mortality as a result of lockdowns. We now refer back to these papers in the discussion on pg. 35 as part of a consideration of possible mechanisms, to wit:

“While we are unable to account for all mechanisms directly in our analysis, such as the burden of home-schooling, our results support the hypothesis that death rates are a major driver of trends in negative affect during conditions of pandemic and lockdown. Changes in death rates and affect mirror each other, broadly speaking, in all the countries we analysed. This suggests that lockdowns work on mood by reducing deaths, as substantiated by studies of excess mortality during COVID [1, 2]. An additional nuance to this view provided by our analysis is that the mood effects were most pronounced among the elderly, who were not affected by economic supports but were most at risk from COVID. During the first month of the pandemic, the proportion of young people (18–24) who reported feeling ‘scared’ during the past week to YouGov rose by 10 percentage points, whereas among the older respondents (65+) this figure was 19 percentage points. Lockdowns reduced deaths in this older demographic and, by association, reduced feelings of fear among this demographic and their loved ones.”

**3 - I think the strongest counterargument to the narrative of the paper is the hedonic adaptation effect…I don’t believe this mechanism because the data is strong in showing how, in each instance, death rates dropped with lockdown and affect followed…I do appreciate the authors using a new method to control for hedonic adaptation by controlling for the lagged DV…yet, is this really adequate to control for hedonic adaptation? … In the papers cited, the controls are for lagged values of the independent variable (not DV). I can’t offer much insights into what the authors should do methodologically but I think they should be careful in this method/argument and triple check and explain how exactly that this method indeed controls for hedonic adaptation.**

Thanks for flagging this. Our estimation strategy was based on a suggestion from Andrew Oswald, who has written many of the seminal papers in the adaptation literature. In response to your concerns, we also reached out to Paul Frijters at the LSE, who has written similarly seminal papers on this subject. He concurred with your point that the standard practice is to use lags of the independent variable that respondents are adapting to. This is appropriate when that independent variable is an event, such as divorce or bereavement. However, it is unsuitable in our case because the independent variables we are interested in, namely the pandemic and lockdowns, are ongoing over the course of our time series, and varying in intensity. As such, using a lag of the dependent variable to estimate the extent of adaptation is more appropriate. Paul noted that this he and coauthors have taken this approach in a recent paper, Etile et al. (2021), just published in the *Journal of Economic Behavior and Organization*, so we have changed the citation in text to this paper (see Citation 51). We have amended the text in the paper to reflect these points (see pg. 24).

**4 - Some of the new analysis raise new questions, especially the split between pre-pandemic and post-pandemic in models 1-3 and 4-6 in the results. I present the questions that they raise below, it would be helpful to provide more answers to these questions in the manuscript.**

• **How is it that there was a period without the pandemic but with lockdowns? From July 19 till Feb 2020. Didn’t the lockdowns start only after the pandemic?**

New Zealand and Australia both had lockdowns during periods of no deaths and very little transmission. See pg. 8 and 34.

**• What is the purpose of this distinction? What value does it add to the paper? “Using two sampling windows in this way illuminates how the association between lockdown and mood differs statistically depending on whether it is assessed relative to a pandemic-free world (models 1-3) or to a world with pandemics but no lockdown (models 4-6).”**

Models 4-6 use only data starting from February 2020, i.e. the start of the pandemic. So the lockdown effect is in comparison to both a) the initial period prior to the first wave of lockdowns but after a pandemic was declared (March 2020) and b) periods of lockdown easing in between and following major national lockdowns. Models 1-3 use the full time-series, including observations prior to February 2020. So here the estimation of the lockdown effect is not just with respect to a) and b) above, but also c) the pre-pandemic period. We are trying to estimate how badly affected people are by lockdowns in the context of a policy response to pandemic outbreak. In this context, it is illuminating to estimate how people’s mood reacts to lockdown when the counterfactual is ‘normal conditions’ and when it is ‘pandemic outbreak’.

**• It is unclear what authors mean in this sentence and why we need this finding/approach, please explain in the manuscript: “The coefficient for being in lockdown is not statistically significant if the sample frame is restricted to the pandemic period, as in models 4-6. This suggests that the result in models 1-3 is driven by comparison to a pandemic-free world.” Please include more explanations and justifications for this analysis and findings.**

Thanks for flagging this as unclear. In addition to the explanation above, we have added the following text to the paper:

“Lockdowns, however, are instituted in response to pandemic outbreak, so a pandemic-free world is arguably an unreasonable comparison case for the effect of lockdown. When the more reasonable comparison case of a pandemic outbreak is used, lockdowns do not seem to have deleterious impacts, broadly speaking.”

**• How does the evidence for youth support the hypothesis as indicated by the following sentence – if anything, it reads like the result for youth is calling the hypothesis into question and requires more explanation of why the results don’t hold for youth: “In contrast, the trend in affect for young (18-24) survey respondents is basically flat despite this demographic being, intuitively at least, the most perniciously a affected by lockdowns. This lends further credence to our hypothesis that we should predict declines in the general population’s affect with pandemic outbreaks and improvements following lockdowns introduced in response to those outbreaks.”**

Thanks for flagging this; we can see the issue. We amended the text to clarify, pg. 31:

“This lends further credence to our hypothesis that the depressed subjective wellbeing observed by studies comparing pre-pandemic and post-lockdown survey responses is driven by the deleterious effects of the pandemic rather than those of lockdowns. If lockdowns were exacerbating negative mood, we would see a more pronounced decline among young people. Our results suggest that we should predict declines in the general population’s affect with pandemic outbreaks and improvements following lockdowns introduced in response to those outbreaks.”

**Minor comments:**

Thank you for these!

**• Page 2: replace comma with period after “introduced,”**

Good catch thanks.

**• Page 3: Unclear what the following sentence means with easing timetables: “where easing timetables were maintained, despite the onset of a new coronavirus wave (such as the United States in the summer of 2020, and the United Kingdom in summer 2021).”**

Oh yep; hopefully the new phrasing is clearer?:

“…and instances where lockdowns were eased according to original schedules despite the onset of a new coronavirus wave”

**• Figure 1 – is it possible to indicate the onset of the pandemic in the graph?**

A good suggestion that we have put into practice.

**• The weighting of the affect states is very clear now and the value and benefits of this method is well-explained.**

Thanks!

**• Could the authors put some numbers to the following comment? How was this conclusion reached? What are the sizes and statistical significance of these changes? “Taking all negative affect items together, negative affect rose sharply with the outbreak of the pandemic, and then continued to rise, albeit much more slowly, after the imposition of lockdown.**

Thanks for flagging this. We have added the following text, pg. 12, which hopefully makes the paper stronger.

“Immediately prior to the pandemic (February 2020), the average negative mood state response among respondents (across all negative mood states) was 23.8%. This rose to 27.6% in March and 30.1% in April, 2020. The pre/post pandemic increase is statistically significant at the p < 0.000 level.”

**• Is it possible to put any statistics to this claim – what is the size of this difference: “These countries also saw a much reduced spike in negative affect during their 2020 lockdowns in comparison to countries that experienced wide scale national epidemics.”**

Thanks for flagging. We have added a brief note to the section on page 21:

“For example, in Australia negative affect rose to a peak of 2.5 times the pre-pandemic baseline during the first lockdown in April 2020. In comparison, in the United States the negative affect index reached 3.3 times and in the United Kingdom 4.3 times the pre-pandemic baseline.”

Some statistics are also provided in the discussion on pg. 33:

"During the first wave of the global coronavirus pandemic, we observe that the increase in negative affect in New Zealand – a country that implemented lockdown without widespread community transmission – never rose more than 1.9 standard deviations above the pre-pandemic mean. Whereas in Great Britain, where a nationwide epidemic led to a significant number of COVID-19 fatalities, negative affect spiked to 4.3 standard deviations above baseline (p < 0.002). Similar patterns can be observed in Australia and the US, which had similar experiences to New Zealand the UK respectively, with affect worsening over the course of pandemic outbreaks but not over the course of lockdowns."

**• In Fig 4, the affect changes in the second and third lockdowns seems smaller than the firsts. In Canada and Ireland, hard to observe a decline in affect the second and third lockdowns on later. This may be worth mentioning and explained.**

Yes that’s an important observations. We now note this on pg. 21 when reporting the descriptive results.

**• Table 2: within the table, can the authors indicate “Pandemic free period” for models 1-3 and “Pandemics but no lockdown” in models 4-6. Otherwise, hard to follow the results.**

Thanks for this suggestion, which we have incorporated.

**• Table 2: please put coefficient of interest in the top row, so it is the first coefficient readers can see and follow.**

Another good suggestion. We have amended the order of variables to the order in which they appear in the subsequent text.

**• Page 24 – 25: In the first paragraph after Table 2, when describing the results, please direct readers to the right column or model. This could be achieved by putting column names in parentheses (Column 1) for the corresponding model/result. Otherwise, hard to follow the results.**

We’ve altered the text as you suggested so it is hopefully easier to follow now.

**• Better to detach these two sentences to avoid confusion--- it reads as if the first sentence summarizes the second (which wouldn’t be correct), but it becomes clear later that the first sentence summarizes the full paragraph: Second, lockdown is statistically associated with a reduction in negative affect. In models 1-3, the dummy variable for being in lockdown has a large, positive, and significant correlation with negative affect.**

Ah yep, that’s fair, thanks. We have amended the text.

**• The discussion and limitation section currently includes only limitations. Please provide an overall discussion of the findings and explain how the findings relate to or complement existing literature.**

Thanks for flagging this. We have added a new paragraph added, as discussed above

**• Page 32: There is a period after “control for”:**

Good catch thanks.
